# Genome-wide analysis of circular RNAs and validation of hsa_circ_0086354 as a promising biomarker for early diagnosis of cerebral palsy

**DOI:** 10.1186/s12920-022-01163-6

**Published:** 2022-01-21

**Authors:** Yuanyuan Hu, Xuzhao Bian, Chao Wu, Yan Wang, Yang Wu, Xiaoqin Gu, Suyan Zhuo, Shiquan Sun

**Affiliations:** 1 Medical College, Xijing University, Xi’an, 710199 Shaanxi People’s Republic of China; 2 School of Public Health, Xi’an Jiaotong University, No.28 Xianning West Road, Beilin District, Xi’an, 710049 Shaanxi People’s Republic of China; 3 Department of Medical Record Management, People’s Hospital of Shapingba District, Chongqing, 400030 People’s Republic of China; 4 Children’s Rehabilitation Department, Xi’an International Medical Center Hospital, Xi’an, 710100 Shaanxi People’s Republic of China; 5 Department of Medical Quality Management, Xi’an International Medical Center Hospital, Xi’an, 710100 Shaanxi People’s Republic of China

**Keywords:** Cerebral palsy diagnosis, Biomarker, hsa_circ_0086354

## Abstract

**Background:**

Cerebral palsy (CP) is a spectrum of non-progressive motor disorders caused by brain injury during fetal or postnatal periods. Current diagnosis of CP mainly relies on neuroimaging and motor assessment. Here, we aimed to explore novel biomarkers for early diagnosis of CP.

**Methods:**

Blood plasma from five children with CP and their healthy twin brothers/sisters was analyzed by gene microarray to screen out differentially expressed RNAs. Selected differentially expressed circular RNAs (circRNAs) were further validated using quantitative real-time PCR. Receiver operating characteristic (ROC) curve analysis was used to assess the specificity and sensitivity of hsa_circ_0086354 in discriminating children with CP and healthy controls.

**Results:**

43 up-regulated circRNAs and 2 down-regulated circRNAs were obtained by difference analysis (fold change > 2, *p* < 0.05), among which five circRNAs related to neuron differentiation and neurogenesis were chosen for further validation. Additional 30 pairs of children with CP and healthy controls were recruited and five selected circRNAs were further detected, showing that hsa_circ_0086354 was significantly down-regulated in CP plasma compared with control, which was highly in accord with microarray analysis. ROC curve analysis showed that the area under curve (AUC) to discriminate children with CP and healthy controls using hsa_circ_0086354 was 0.967, the sensitivity was 0.833 and the specificity was 0.966. Moreover, hsa_circ_0086354 was predicted as a competitive endogenous RNA for miR-181a, and hsa_circ_0086354 expression was negatively correlated to miR-181a expression in children with CP.

**Conclusion:**

Hsa_circ_0086354 was significantly down-regulated in blood plasma of children with CP, which may be a novel competent biomarker for early diagnosis of CP.

**Supplementary Information:**

The online version contains supplementary material available at 10.1186/s12920-022-01163-6.

## Background

Since W.J. Little first described in the 1840s, the concept of cerebral palsy (CP) has been revised for several times and is now defined as a non-progressive motor disorder induced by brain injury during prenatal (80%), perinatal (10%) or postnatal (10%) [1, 2]. The incidence of CP is 1.25 per 1000 neonates in China and 2–3 per 1000 neonates worldwide [3, 4]. The brain injury in children with CP results in activity limitation in most cases, accompanying with impaired communication and cognition [5, 6]. To date, CP has no cure and would cost millions of healthcare expenditure, making CP as a severe public health problem that brings enormous burden for patient families [2, 7]. Preterm birth and asphyxia result from dystocia are the most common risk factors for CP [8, 9]. Administration of magnesium sulfate for women at risks of premature delivery and cooling therapy for infants at high risks of CP are considered to be effective preventive methods [10–12]. Unfortunately, existing diagnosis by comprehensive analysis of neonatal encephalopathy history, neuroimaging and neurodevelopmental assessment is limited and needs further researches [4, 13]. Therefore, a better understanding of CP aetiology and pursuit of more accurate early diagnostic methods are of great importance.

Noncoding RNAs represents more than 98% of all human transcripts, among which circular RNAs (circRNAs) are a special subtype without 5′ cap or 3′ poly-A tail [14, 15]. circRNAs become a new research hotspot in the past decade owing to their diverse physiological functions: circRNAs sponge microRNAs according to the “competing endogenous RNA” (ceRNA) [16]; they also act as protein scaffolds or templates for protein translation [17, 18]. Besides, increasing evidence indicates that circRNAs are implicated in the regulation of various human diseases including cardiovascular diseases, cancers and neurological diseases [19–21]. circRNAs may also serve as potent biomarkers for the early detection of specific diseases attributing to its stability and easy accessibility [22, 23].

With the rapid development of next-generation sequencing, over 1000 circRNAs in human serum exosomes were identified [23, 24]. In present study, we screened out differential expressed circRNAs between children with CP and their healthy controls using microarray technology, to select novel biomarkers for early diagnosis and intervention of CP as well as provide a better understanding of CP etiology.

## Methods

### Sample preparation

Five children with CP and their healthy twins were selected in our study to minimize individual differences (Average age: 3.3 ± 1.5, average birth weight: 2.9 ± 0.4 kg). Detailed clinical information of participants was provided in Additional file 1: Table S1. Additional 30 pairs of children with CP and healthy controls (without any congenital or acquired disease) were recruited for subsequent validation of differential expressed circRNAs (Age: 4.2 ± 1.6; average birth weight: 3.1 ± 0.6 kg). The diagnostic criterion for CP were the combination of following clinical findings: ① motor dysfunction assessed using the Hammersmith Infant Neurological Examination (HINE); ② abnormal neuroimaging detected by magnetic resonance imaging (MRI); ③ comprehensive assessment of clinical history and high risks for CP including prematurity and low birthweight [13, 25]. Inclusive criteria: ①undergo no drug therapy; ② with complete clinical information; Exclusive criteria: ① acute/chronic infectious diseases, connective tissue diseases or malignant tumor; ② recent use of immunosuppressant; ③ injury of liver and kidney function. Whole blood sample (5 ml per patient) was collected in Na_2_EDTA tubes. Plasma was isolated by centrifugation, followed by total RNA extraction using TRIzol reagent (ThermoFisher Scientific, Waltham, MA, USA). All blood samples were collected with the consent of parents of children with CP. And all experiments performed in this study were in accord with the ethical guidelines of the Declaration of Helsinki and approved by the Ethics Committee of Xi’an International Medical Center Hospital.

### Microarray analysis

After RNA integrity assessment using Agilent Bioanalyzer 2100 (Agilent technologies, Santa Clara, CA, USA), total RNAs were reversely transcribed into cDNA, which was further used to generate biotinylated cRNAs. Then cRNAs were hybridized with Hybridization Slides (Agilent technologies, Santa Clara, CA, USA) in a Hybridization Oven at 65 °C for 17 h. Sides were scanned under a Microarray Scanner (Agilent technologies, Santa Clara, CA, USA) and raw data were obtained by the Feature Extraction software 10.7 (Agilent technologies, Santa Clara, CA, USA), followed by raw data normalization using Quantile algorithm. cirRNAs with a fold change > 2, *p* value < 0.05 were presented as heatmap plots using R package “pheatmap”. Then differential expressed circRNAs with flag-signal of “Absent” in CP group or healthy controls were removed. Gene Ontology (GO) enrichment analysis were performed use Fisher's exact test by a R package “clusterProfiler” of the target genes. For CP etiology and biomarker investigations, five circRNAs regarding neuron differentiation and neurogenesis were chosen for further quantitative real-time PCR verification. The TargetScan prediction tool was used to identify interactions between hsa_circ_0086354 and target miRNAs. miRNAs that had perfect nucleotide pairing with hsa_circ_0086354 were selected. Further Pearson correlation was carried out to analyze the correlation between hsa_circ_0086354 and miRNAs, only interactions with significant negative correlation was retained. The circRNAs-miRNAs network was visualized by Cytoscape software (version 3.7.0; http://www.cytoscape.org) [26].

### Quantitative real-time PCR

Additional thirty pairs of children with CP and their healthy controls were recruited to verify the differential expressed circRNAs screened by the microarray. In brief, total RNAs of plasma were extracted using UNIQ-10 RNA extraction kit (Sangon Biotech, Shanghai, China) and reversely transcribed into cDNA using Maxima Reverse Transcriptase (ThermoFisher Scientific, Waltham, MA, USA). Then cDNAs were quantified using Fast qPCR Master Mix (High Rox) (Sangon Biotech, Shanghai, China) in an ABI Stepone plus PCR instrument. Similar methods were used to detect miR-181a level. 18S ribosomal RNA was used as internal control for hsa-circRNAs and RNU6B was used as an internal control for miR-181a. All data were analyzed using the 2^−△△^ method. Specific primers used for circRNAs detection were listed in Table 1.

**Table 1:** Primers used for quantitative real-time PCR in this study

Target	Primers
hsa_circ_0042123	Forward: 5′-TCAGCAACAGGAGGAGCATT-3′
	Reverse: 5′-CCTCAGGAAATGTCCACCACT-3′
hsa_circ_0083264	Forward: 5′-AAGCCCATCCAGAGGTTCC-3′
	Reverse: 5′-CTGTTCTCCCTCTTCCTCTTCAT-3′
hsa_circ_0035127	Forward: 5′-TCTATTCATTCCTCCAAAACCTG-3′
	Reverse: 5′-ATGGGAAGCGGAATGAGAG-3′
hsa_circ_0086354	Forward: 5′-ACTTGGGCTGGTGCAACTAA-3
	Reverse: 5′-GGCCCGGGCCATATAGT-3′
hsa_circ_0015069	Forward: 5′-ACTCGCAGCCAGTCAGATGTA-3′
	Reverse: 5′-TGACTGCACGCTCATGAACA-3′
Hsa-18s rRNA	Forward: 5′-GGACACGGACAGGATTGACA-3′
	Reverse: 5′-CCAGAGTCTCGTTCGTTATCG-3′
miR-181a	Forward: 5′-TGTGATGTGGAGGTTTGC-3′
	Reverse: 5′-AGTCCTGGTGTGTCCA-3′
RNU6B	Forward: 5′-CTCGCTTCGGCAGCACA-3
	Reverse: 5′-TGGTGTCGTGGAGTCG-3′

### Statistical analysis

Data from quantitative real-time PCR was analyzed using the 2^−△△^ method and mean values were compared using unpaired t-test (Graphpad Prism 8.0, USA). All experiments were repeated for at least three times and *p* values less than 0.05 were regarded as statistically significant.

## Results

### Differentially expressed circRNAs screened by microarray

**Fig. 1:**
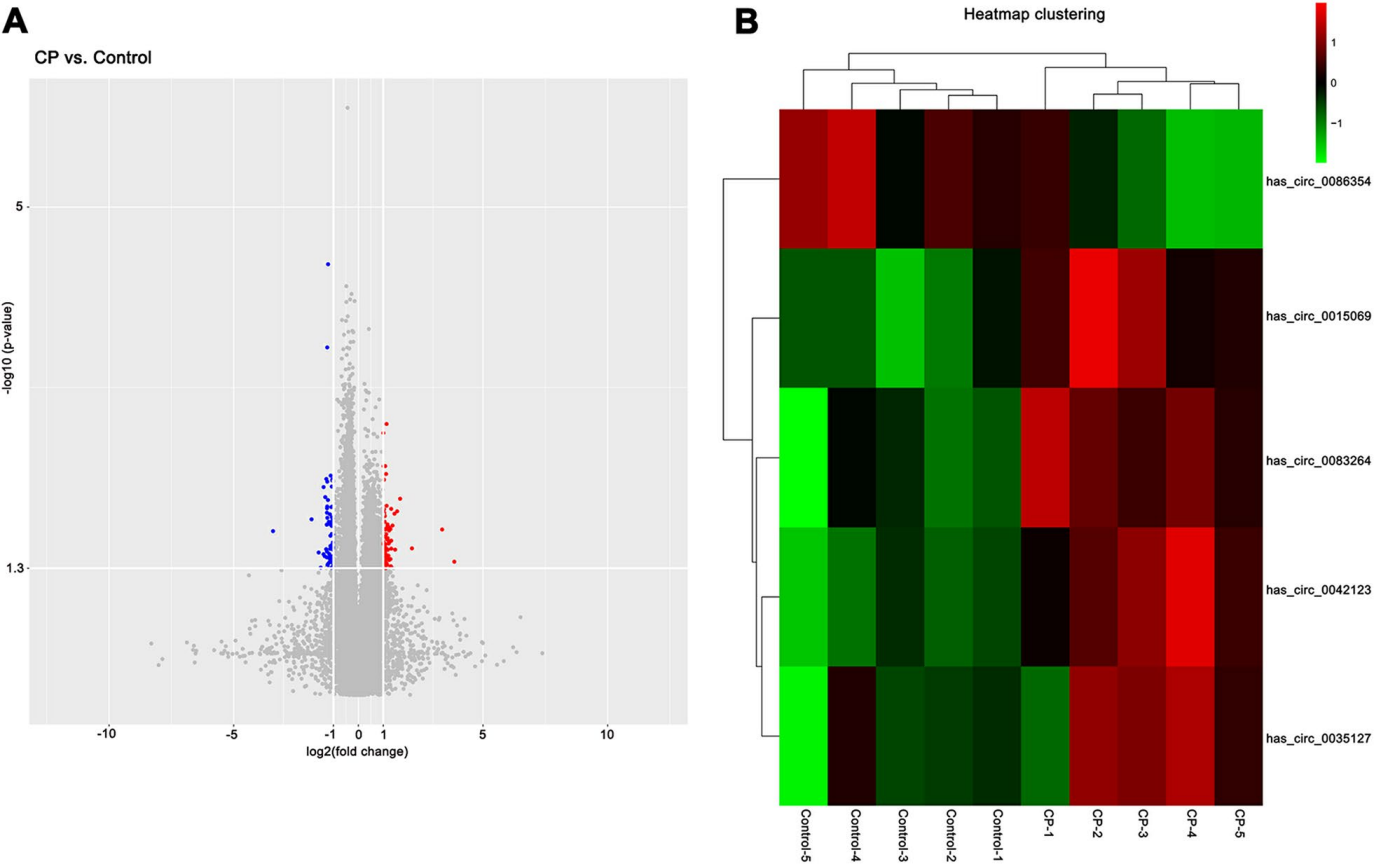
Differentially expressed circRNAs between children with CP and their healthy twins. **A** Differentially expressed circRNAs between children with CP and their healthy controls was shown in Volcano plot. “Red” represents up-regulated circRNAs, “Blue” represents down-regulated circRNAs (fold change > 2, *p* < 0.05). **B** Heatmap clustering analysis was performed to display 5 selected circRNAs. Rows represent differential circRNAs and columns represent five pairs of CP samples and healthy controls. “Green” represents down-regulation and “Red” represents up-regulation of circRNAs in each sample. *CP* cerebral palsy

In order to minimize individual differences, the blood samples from five pairs of twin children (one CP and one healthy) were collected in our study. Sino human ceRNA array V3.0 which includes 53,625 human circRNAs was used to screen out differentially expressed circRNAs between the twins. Volcano plot showed that 134 circRNAs were differentially expressed in children with CP compared to their healthy controls, among which 77 circRNAs were up-regulated and 57 were down-regulated (fold change > 2, *p* < 0.05) (Fig. 1A). After further flag-signal screening, 45 differentially expressed human circRNAs were obtained (fold change > 2, *p* < 0.05). As listed in Table 2, 43 circRNAs were up-regulated and 2 circRNAs were down-regulated. According to GO enrichment analysis, we selected five differentially expressed circRNAs that were mainly involved in neuron differentiation and neurogenesis for further quantitative real-time PCR verification (Additional file 2: Fig. S1). As clustering analysis of heatmap shows, hsa_circ_0042123 (host gene: peripheral myelin protein 22 (PMP22)), hsa_circ_0083264 (host gene: Rho guanine nucleotide exchange factor 10 (ARHGEF10)), hsa_circ_0035127 (host gene: myelin expression factor 2 (MYEF2)) and hsa_circ_0015069 (host gene: PBX homeobox 1 (PBX1)) were up-regulated in CP group while hsa_circ_0086354 (host gene: protein tyrosine phosphatase receptor type D (PTPRD)) was down-regulated versus the control (Fig. 1B).

**Table 2:** Microarray analysis of differential expressed circRNAs in 5 CP children compared with their healthy twins

CircRNA_ID	Regulation	Fold change	*p* values	Circ_chromosome	Host gene
hsa_circ_0062733	Up	3.195	0.010	chr22	EMID1
hsa_circ_0066747	Up	2.496	0.032	chr3	MYH15
hsa_circ_0030588	Up	2.492	0.012	chr13	ABCC4
hsa_circ_0020792	Up	2.488	0.048	chr11	INS-IGF2
hsa_circ_0007110	Up	2.466	0.048	chr9	DENND4C
hsa_circ_0049906	Up	2.442	0.040	chr19	HAUS8
hsa_circ_0031700	Up	2.391	0.027	chr14	MIPOL1
hsa_circ_0036358	Up	2.360	0.027	chr15	PTPN9
hsa_circ_0036730	Up	2.350	0.021	chr15	C15orf42
hsa_circ_0066990	Up	2.319	0.028	chr3	KPNA1
hsa_circ_0016754	Up	2.267	0.032	chr1	CDC42BPA
hsa_circ_0068412	Up	2.257	0.025	chr3	IGF2BP2
hsa_circ_0087881	Up	2.208	0.025	chr9	CTNNAL1
hsa_circ_0084683	Up	2.186	0.039	chr8	CSPP1
hsa_circ_0042530	Up	2.182	0.031	chr17	POLDIP2
hsa_circ_0035047	Up	2.147	0.020	chr15	WDR76
hsa_circ_0035127	Up	2.144	0.050	chr9	MYEF2
hsa_circ_0043970	Up	2.136	0.044	chr17	NBR1
hsa_circ_0054449	Up	2.133	0.020	chr2	EPAS1
hsa_circ_0068411	Up	2.131	0.036	chr3	IGF2BP2
hsa_circ_0071500	Up	2.128	0.039	chr4	WWC2
hsa_circ_0033776	Up	2.126	0.038	chr14	None
hsa_circ_0084682	Up	2.123	0.036	chr8	CSPP1
hsa_circ_0071499	Up	2.120	0.024	chr4	WWC2
hsa_circ_0087309	Up	2.118	0.031	chr9	TLE1
hsa_circ_0090182	Up	2.117	0.033	chrX	PRRG1
hsa_circ_0036485	Up	2.114	0.042	chr15	ADAMTS7
hsa_circ_0015069	Up	2.113	0.004	chr1	PBX1
hsa_circ_0013249	Up	2.087	0.030	chr1	TMEM56
hsa_circ_0087882	Up	2.082	0.017	chr9	CTNNAL1
hsa_circ_0071976	Up	2.078	0.040	chr5	ANKH
hsa_circ_0087880	Up	2.077	0.043	chr9	CTNNAL1
hsa_circ_0009100	Up	2.055	0.014	chr17	PRR11
hsa_circ_0030584	Up	2.051	0.024	chr13	ABCC4
hsa_circ_0039989	Up	2.046	0.012	chr16	CDH3
hsa_circ_0083264	Up	2.039	0.006	chr8	ARHGEF10
hsa_circ_0056717	Up	2.036	0.024	chr2	RIF1
hsa_circ_0045000	Up	2.024	0.029	chr17	BCAS3
hsa_circ_0047155	Up	2.015	0.028	chr18	RIOK3
hsa_circ_0087884	Up	2.013	0.027	chr9	CTNNAL1
hsa_circ_0016274	Up	2.007	0.022	chr1	YOD1
hsa_circ_0042123	Up	2.003	0.002	chr17	PMP22
hsa_circ_0062335	Up	2.002	0.029	chr22	PI4KA
hsa_circ_0077792	Down	0.492	0.027	chr6	TRMT11
hsa_circ_0086354	Down	0.272	0.016	chr15	PTPRD

### Hsa_circ_0086354 is a potential biomarker for early diagnosis of CP

**Fig. 2:**
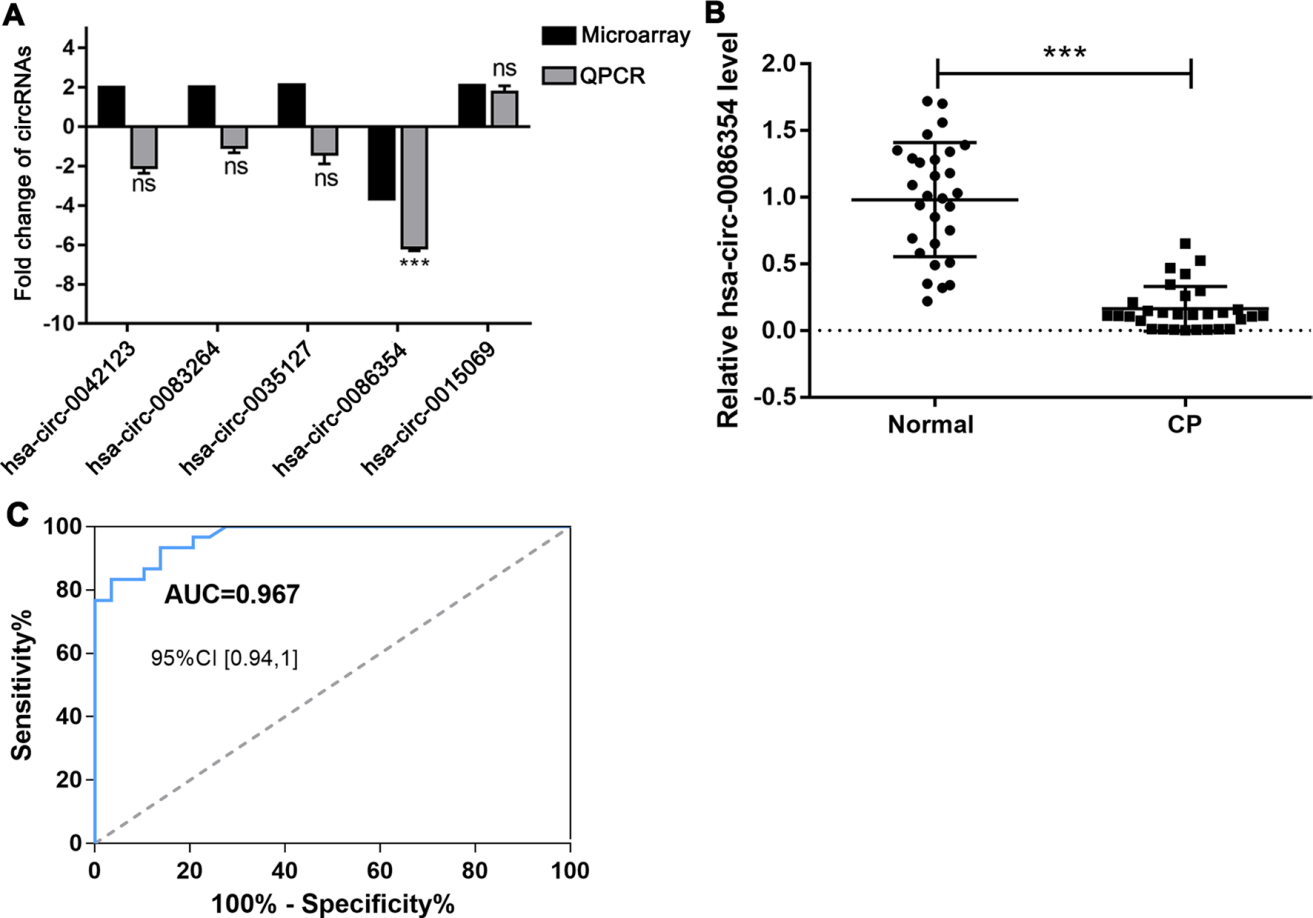
Hsa_circ_0086354 is a potential biomarker for early diagnosis of CP. Five circRNAs screened out by microarray was further validated by quantitative real-time PCR. **A** All expression levels in Control group were normalized to “1”. Fold changes of CP versus Control obtained from microarray and real-time PCR were shown. **B** Relative expression of hsa_circ_0086354 in 30 pairs of CP and Control samples detected by quantitative real-time PCR was shown. **C** ROC curve analysis was carried out to assess the value of hsa_circ_0086354 in discriminating children with CP and healthy controls. The AUC was 0.967, the sensitivity was 0.833 and the specificity was 0.966. “ns”: not significant; ***p < 0.001*.* *CP* cerebral palsy, *ROC* receiver operating characteristic, *AUC* area under the curve

Further quantitative real-time PCR validation showed that the fold changes of CP versus Control were as follow: hsa_circ_0042123 was − 2.067 (microarray: 2.003), hsa_circ_0083264 was − 1.031 (microarray: 2.039), hsa_circ_0035127 was − 1.408 (microarray: 2.144), hsa_circ_0015069 was 1.76 (microarray: 2.113) and hsa_circ_0086354 was − 6.15 (microarray: − 3.676) (Fig. 2A). The expression pattern of hsa_circ_0086354 validated by real-time PCR was highly in accord with that detected by microarray, showing that hsa_circ_0086354 was significantly down-regulated in CP group (Fig. 2B). Further receiver operating characteristic (ROC) curve analysis showed that the area under the curve (AUC) to discriminate CP and healthy controls using hsa_circ_0086354 level was 0.967, the sensitivity was 0.833 and the specificity was 0.966 (Fig. 2C), suggesting that hsa_circ_0086354 is a potential biomarker for CP diagnosis.

### miR-181a is a downstream target of hsa_circ_0086354 in CP

**Fig. 3:**
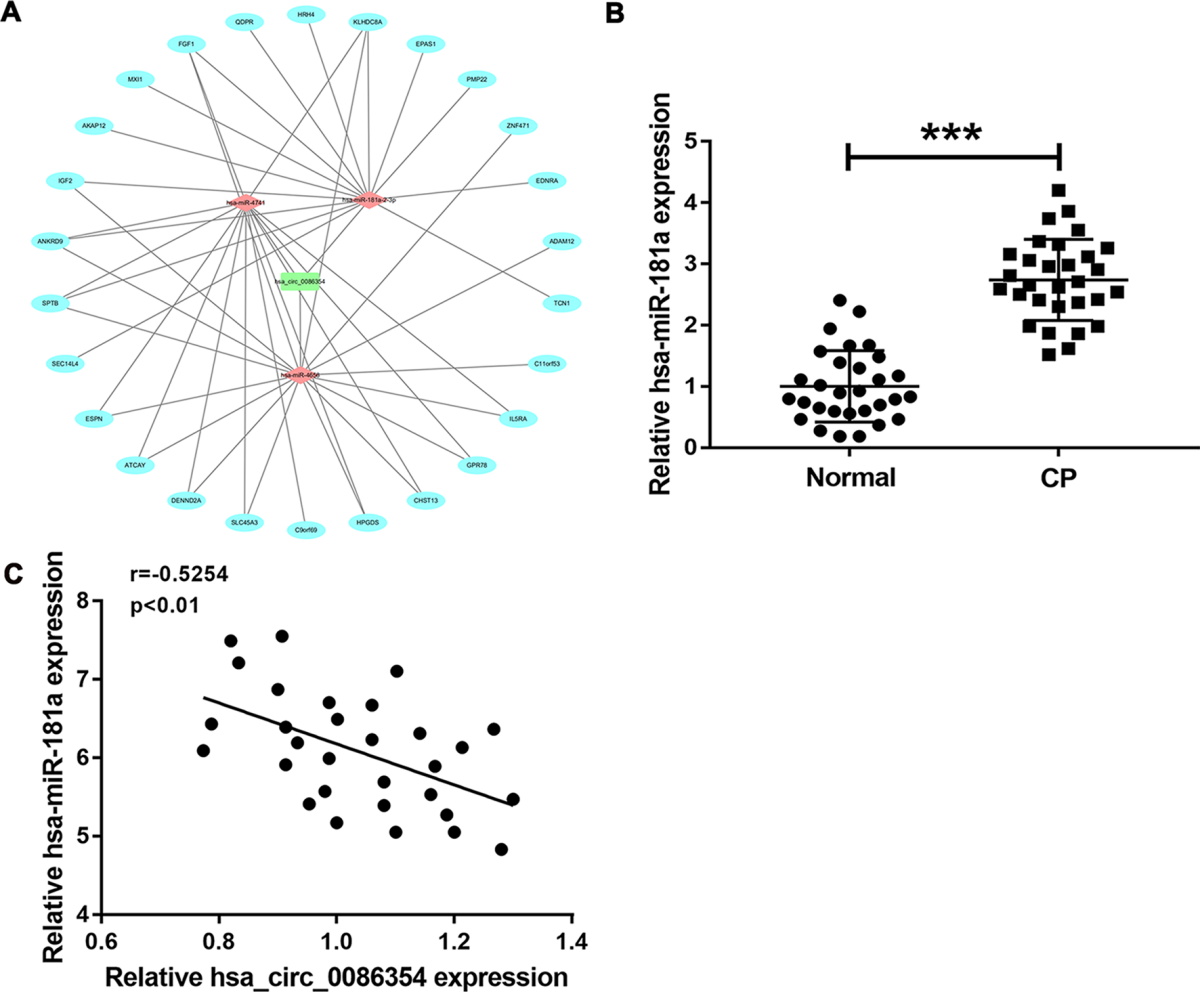
miR-181a is a downstream target of hsa_circ_0086354 in CP. **A** Cytoscape analysis was performed to show the hsa_circ_0086354 associated ceRNA network. **B** The expression of miR-181a was detected using quantitative real-time PCR. **C** The correlation between hsa_circ_0086354 expression and miR-181a expression was analyzed using Graphpad Prism 8.0. *ceRNA* competitive endogenous RNA, *CP* cerebral palsy

Hsa_circ_0086354 associated ceRNA network was obtained using Cytoscape analysis. miR-181a, miR-4741 and miR-4656 were down-stream target microRNAs of hsa_circ_0086354 (Fig. 3A). Further quantitative real-time PCR assay showed that miR-181a level was significantly up-regulated in children with CP (Fig. 3B). Besides, the miR-181a level was negatively correlated to hsa_circ_0086354 level in children with CP (Fig. 3C). These results implied that miR-181a is a downstream target of hsa_circ_0086354 in CP.

## Discussion

Owing to its enigmatic etiology, the diagnosis of CP can barely rely on neuroimaging and assessment of motor dysfunction [27]. CirRNAs were first considered as byproducts of mis-splicing, yet increasing evidence indicated that circRNAs are implicated in various molecular processes as well as human diseases: circRNAs regulate gene expression via regulating gene transcription, gene splicing or sponging microRNAs; circRNAs are involved in the regulation of neuronal diseases, cardiovascular disease and cancer progression. Of note, ciRS-7 regulates α-synuclein expression through co-expressing and co-localizing with miR-7 to further regulate brain development [24]. Besides, majority of identified circRNAs are abundantly detected in brain tissues and neurons, which inspired us to explore specific biomarkers for CP diagnosis.

In the present study, blood samples from five children with CP and their twin brothers/sisters were collected to screen out differentially expressed circRNAs using microarray. Twin participants at identical preterm conditions can exclude additional risk factors of CP, which makes our results more reliable. Five circRNAs enriched in neuron differentiation and neurogenesis were selected from 45 differentially expressed circRNAs for further validation. Another 30 pairs of plasma samples from children with CP and healthy controls were collected, and the expression levels of five selected circRNAs were quantified. It was remarkable that the expression pattern of hsa_circ_0086354 measured by quantitative real-time PCR was highly in consistent with that detected by microarray. Yet the expression differences between children with CP and healthy controls of hsa_circ_0042123, hsa_circ_0083264, hsa_circ_0035127 and hsa_circ_0015069 were either not significant or contradictory with microarray analysis. Therefore, our findings suggest that hsa_circ_0086354 might serve as a promising biomarker for CP diagnosis.

circRNAs have been reported to serve as competent biomarkers for diagnosis of various diseases. For instance, plasma hsa_circRNA_002453 was a potential biomarker for severity of renal involvement and diagnosis of lupus nephritis with an AUC of 0.906 [28]. Hsa_circRNA_0000520 is remarkably down-regulated in gastric cancer and may serve as a potential biomarker for early diagnosis [29]. Hsa_circRNA_0001649 is a novel specific biomarker for colorectal cancer assessment [30]. circRNAs display high stability owing to their covalent loop structure, which helps them get rid of de-adenylation, de-capping and RNases degradation. The tissue-specific expression pattern of circRNAs enables them to serve as specific biomarkers for specific diseases [31, 32]. The application of circRNAs as biomarkers has always been a controversial topic, and the abundance of circRNAs is the major concern. Indeed, generally, the abundance of circRNAs is relatively low compared to their linear RNA product in body fluids. However, others demonstrated that some circRNAs are detected at comparable, even higher expression to their linear RNA [33, 34]. Besides, the rapid development of next-generation sequencing will provide substantial technical support for circRNA detection. Dong, R concluded that majority of annotated circRNAs are identified in brain tissues and neurons [35]. In the present study, hsa_circ_0086354 was significantly down-regulated in CP plasma with an AUC of 0.967, suggesting hsa_circ_0086354 may be a promising biomarker for the early diagnosis of CP. In addition, the host gene of hsa_circ_0086354 is PTPRD, which is highly expressed in brain tissues and regulated neurite growth and neurons axon guidance, indicating that PTPRD and hsa_circ_0086354 might involve in CP etiology [36, 37].

We further discovered that hsa_circ_0086354 acts as a ceRNA of miR-181a. miR-181a is up-regulated in patients with mild cognitive impairment which later progressed to Alzheimer’s disease [38]. miR-181a is also up-regulated in rats after ischemia/reperfusion induced cerebral injury [39]. On the contrary, miR-181a silencing exerts neuroprotective effects through suppressing neuronal apoptosis and neuronal loss both in a rat model and in epilepsy children [40, 41]. MiR-181a silencing also promotes neuronal growth via regulating the Smad signaling in Parkinson’s disease [42]. Besides, miR-181a contributes to neural stem cell differentiation and promotes generation of neurons [43, 44]. Here we found that miR-181a was significantly up-regulated in children with CP, and miR-181a level was negatively correlated to hsa_circ_0086354 level. All these findings imply that hsa_circ_0086354 might be involved in the regulation of neuronal survival and neuronal differentiation through targeting miR-181a.

## Conclusion

Hsa_circ_0086354 is significantly down-regulated in children with CP in contrast with their healthy control with an AUC of 0.967, making it as a promising biomarker for the early diagnosis of CP. Hsa_circ_0086354 may also be involved in the etiology of CP through targeting miR-181a.

## Supplementary Information

**Additional file 1: Table S1.** Relative clinical information of children with cerebral palsy and their healthy controls.

**Additional file 2: Fig. S1.** Top 30 of biological_process, cellular_component and molecular_function obtained using Gene Ontology enrichment. Plot size refers to gene number.

## Data Availability

The datasets generated and/or analysed during the current study are available in the Gene Expression Omnibus (GEO) database at https://www.ncbi.nlm.nih.gov/geo/query/acc.cgi?acc=GSE183021 (accession number: GSE183021).

## Abbreviations

CP: Cerebral palsy
circRNAs: Circular RNAs
ceRNA”: Competing endogenous RNA”
ROC: Receiver operating characteristic
AUC: Under the curve
PTPRD: Protein tyrosine phosphatase receptor type D
