# Supplementary material for: Genome-wide analysis of circular RNAs and validation of hsa_circ_0086354 as a promising biomarker for early diagnosis of cerebral palsy
Source: BMC Med Genomics. 2022 Jan 21;15:13. doi: 10.1186/s12920-022-01163-6 (PMC8783515; doi:10.1186/s12920-022-01163-6)
Supplement: Supplementary file 2 — Additional file 2: Fig. S1. Top 30 of biological_process, cellular_component and molecular_function obtained using Gene Ontology enrichment. Plot size refers to gene number. [file 12920_2022_1163_MOESM2_ESM.docx]

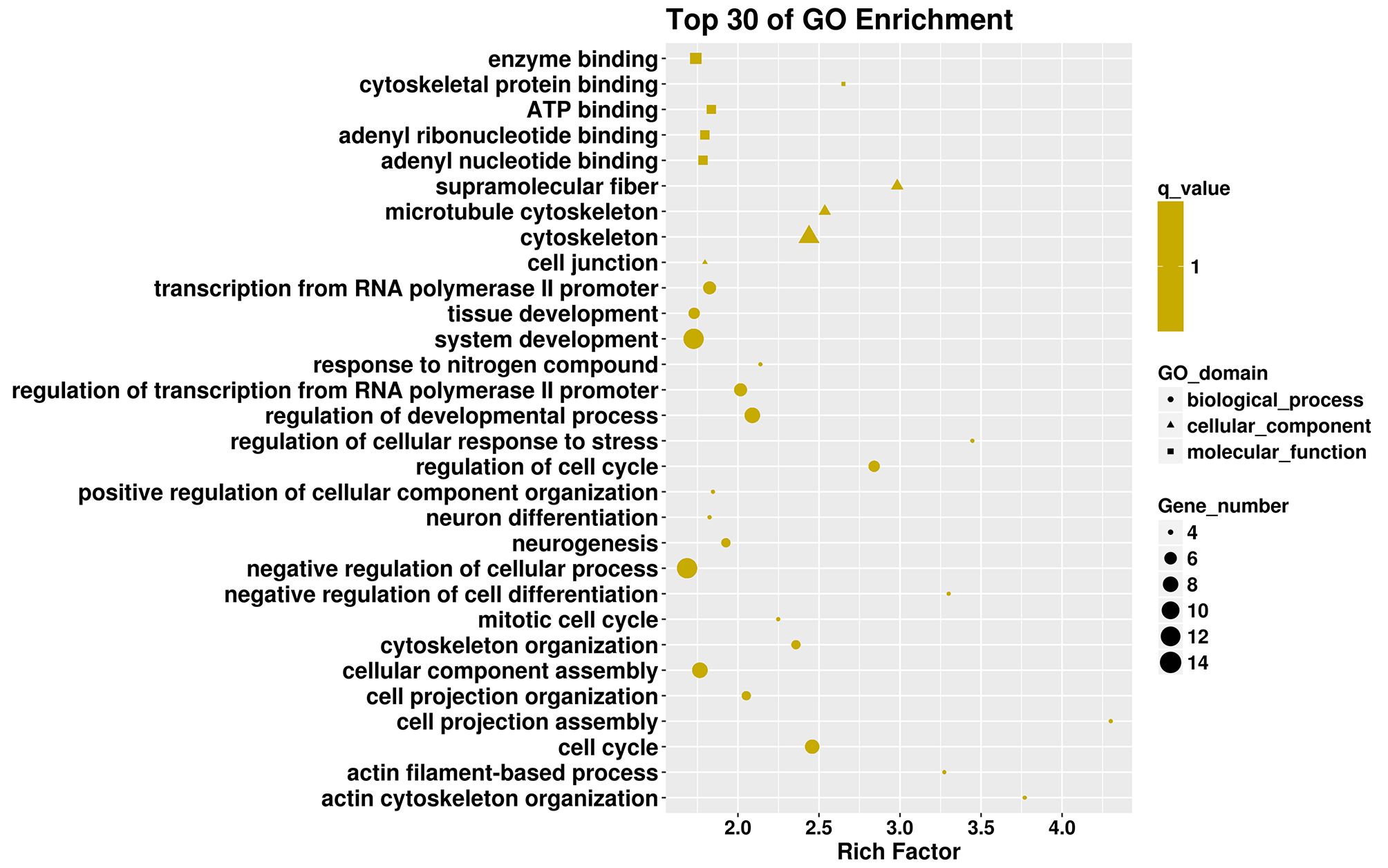
**Supplementary material figure 1.** Top 30 of biological_process, cellular_component and molecular_function obtained using Gene Ontology enrichment. Plot size refers to gene number.
